# Brain Region-Dependent Rejection of Neural Precursor Cell Transplants

**DOI:** 10.3389/fnmol.2018.00136

**Published:** 2018-04-30

**Authors:** Nina Fainstein, Tamir Ben-Hur

**Affiliations:** Department of Neurology, The Agnes Ginges Center for Human Neurogenetics, Hadassah Medical Center, Hebrew University of Jerusalem, Jerusalem, Israel

**Keywords:** hippocampus, rejection, CD200, transplantation, NPCs

## Abstract

The concept of CNS as an immune-privileged site has been challenged by the occurrence of immune surveillance and allogeneic graft rejection in the brain. Here we examined whether the immune response to allogeneic neural grafts is determined by the site of implantation in the CNS. Dramatic regional differences were observed between immune responses to allogeneic neural precursor/stem cell (NPC) grafts in the striatum vs. the hippocampus. Striatal grafts were heavily infiltrated with IBA-1+ microglia/macrophages and CD3+ T cells and completely rejected. In contrast, hippocampal grafts exhibited milder IBA-1+ cell infiltration, were not penetrated efficiently by CD3+ cells, and survived efficiently for at least 2 months. To evaluate whether the hippocampal protective effect is universal, astrocytes were then transplanted. Allogeneic astrocyte grafts elicited a vigorous rejection process from the hippocampus. CD200, a major immune-inhibitory signal, plays an important role in protecting grafts from rejection. Indeed, CD200 knock out NPC grafts were rejected more efficiently than wild type NPCs from the striatum. However, lack of CD200 expression did not elicit NPC graft rejection from the hippocampus. In conclusion, the hippocampus has partial immune-privilege properties that are restricted to NPCs and are CD200-independent. The unique hippocampal milieu may be protective for allogeneic NPC grafts, through host-graft interactions enabling sustained immune-regulatory properties of transplanted NPCs. These findings have implications for providing adequate immunosuppression in clinical translation of cell therapy.

## Introduction

While the brain has been traditionally considered an immune-privileged site, it has become clear that the CNS is not isolated from the immune system. On one side the blood-brain-barrier (BBB) precludes non-specific systemic immune response from penetrating the brain (Medawar, 1948) and there is only partial drainage of interstitial fluid to cervical lymph nodes, excluding antigen-presenting cells (Engelhardt et al., 2016). Conversely, the CSF is regularly patrolled by immune cells (Carson et al., 2006). In addition, accumulating literature suggests that neural cell grafts can be rejected from the CNS (Brundin et al., 1989; Armstrong et al., 2001). Xenogeneic neural stem/precursor cell (NPC)-sphere transplantation to the striatum and cortex resulted in almost complete rejection of grafted cells 2 months post-transplantation (Hicks et al., 2008). Recent work studying the implantation of human induced pluripotent stem cell-derived NPCs (Itakura et al., 2015) showed a complete rejection in the absence of proper immunosuppressive treatment. Moreover, transplantation of similar cells in neonates in order to induce neonatal immune tolerance also ended in graft rejection (Mattis et al., 2014). Nonetheless, human embryonic stem cell-derived cells transplanted in a rat model of Parkinson’s disease were detected 3 months following transplantation (Ben-Hur et al., 2004; Brederlau et al., 2006). We hypothesized that these seemingly-contradictory findings between labs that studied immune-rejection of cell grafts were a result of differences in the immunogenicity of the grafted cells (Armstrong et al., 2001) as well as in the characteristics of the host CNS in different experimental paradigms, in terms of site of engraftment and its state of health.

Various types of stem/precursor cells possess powerful therapeutic immune-regulatory properties. We and others have shown that NPCs inhibit the activation and proliferation of T cells (Einstein et al., 2007; Fainstein et al., 2008) and inhibit antigen presenting cells (Pluchino et al., 2009b). While these properties proved to be clinically significant in inhibiting neuro-inflammation (Einstein et al., 2006b; Bacigaluppi et al., 2009; Pluchino et al., 2009a), the eventual loss of these properties after NPC transplantation into the naïve brain, resulted in their active rejection (Fainstein et al., 2013b). Therefore, the immunogenicity of allogeneic grafts has become a central issue for clinical translation of cell therapy for neurological diseases. In particular, it is crucial for determining the degree of immunosuppression that is required to prevent graft rejection. Furthermore, understanding the complexity and biological basis of graft-induced immune response might help improve the long-term fate of transplanted cells in the brain.

In addition to their immune-regulatory properties, NPC exert powerful neurotrophic and neuroprotective effects. For example, NPC transplantation to the hippocampus has been shown to restore short term memory in several mouse models of memory impairment, including Alzheimer’s disease (Ben Menachem-Zidon et al., 2008, 2015; Ben-Shaanan et al., 2008).

We have recently shown that the survival of syngeneic NPC grafts is dependent in regionally-determined graft-host interactions (Fainstein et al., 2013a). We showed that the hippocampal environment is supportive for transplanted NPC survival, as opposed to significantly reduced survival in the striatum. This notion suggests the hypothesis that the extent of allogeneic graft rejection from the CNS may also depend on region-specific graft-host brain interactions. Specifically, we asked whether the hippocampal milieu may be protective against allogeneic graft rejection. Therefore, we compared the immune response against allogeneic NPC grafts in the striatum and hippocampus and studied the mechanisms mediating the different regionally-determined immune response and consequent graft fate.

We found that while NPC grafts were rejected from the striatum, they persisted in the hippocampus, by precluding T-cell infiltration. Conversely, transplantation of non-stem cell neural cells, namely astrocytes elicited vigorous rejection. NPC rejection in the striatum was associated with loss of expression of the immune-modulatory surface protein CD200 and as expected, CD200-deficient NPC striatal grafts were rejected more rapidly than wild type NPCs. However, transplanted CD200-deficient NPCs were not rejected from the hippocampus. Thus, graft-host interactions mediate the protective property of the hippocampus against its rejection. These interactions are specific to NPCs and CD200-independent.

## Materials and Methods

### Animals

Eleven to 12 weeks old BalB/C female mice were supplied by Harlan and were raised under specific pathogen-free conditions. Animal experimentation was approved by the Hebrew University institutional animal care and use ethics committee, approval no. MD-14-14158-5.

### Isolation and Growth of Mouse NPCs and Primary Astrocytes

Multi-potential NPCs were isolated from the forebrain of embryos on day 13.5 of pregnancy from wild type C57BL/6 mice and two transgenic strains: (1) C57BL/6 expressing green fluorescent protein (GFP); and (2) C57BL/6 CD200KO (Kind gift of Dr. Sedgwick). The tissue was dissociated using EBSS containing 0.25 mg/mL trypsin and 10 μg/mL DNase I (5 min at 37°C). Tissue was further mechanically dissociated by aspiration and expulsion with a 5 mL Falcon pipette. Dissociated cells were plated in T-75 flasks. For generation of NPC spheres, cells were plated (30 × 10^5^ cells/ml) and grown for 5 days in a serum-free DMEM/F-12 medium containing B27 supplement, and basic fibroblast growth factor (FGF2, 10 ng/mL, R&D systems), was added to cultures daily. NPC spheres consisted of 200–300 Nestin+ cells and <1% GFAP+ cells or β-tubulin+ cells.

To obtain purified primary cultures of astrocytes, mixed glial cultures of 1 day old C57BL/6 GFP+ pups were prepared. Following a similar dissociation protocol, forebrain cells were plated (30 × 10^6^ cells/flask) in 10 μg/ml poly-D-lysine (PDL, Sigma-Aldrich) coated T-75 flasks and grown in DMEM medium supplemented with 5% fetal bovine serum (Biological Industries). Astrocytes were enriched from these 7-day-old mixed glial cultures using the shaking method as previously described (Ben-Hur et al., 1998), and then passaged once and cultured for 1 week to obtain confluency and growth arrest. These homogeneous cultures, consisting of >99% GFAP+ cells were used for transplantation.

### Experimental Design: NPCs and Astrocyte Transplantation

Mice were anesthetized using a combination of ketamine (80 mg/kg; i.p.) and xylazine (20 mg/kg; i.p.) prepared in normal saline. Quantities of 2 × 10^3^ NPC spheres or 1 × 10^6^ astrocytes in a volume of 2 μl of F12/DMEM or DMEM were injected into the hippocampus (*A* = −2.2 mm, *L* = 2 mm, *H* = 2.7 mm). Quantities of 5 × 10^3^ NPC spheres were injected into the naïve striatum (*A* = 0.5 mm, *L* = 2 mm, *H* = 4.5 mm). Wild-type and CD200 KO NPC spheres were labeled with 30 μM BrdU (sigma) for 48 h prior to transplantation. In some experiments, mice were treated with daily injection of i.p. cyclosporine A (10 mg/kg) or saline as vehicle. Animals were sacrificed for histopathological analysis (see below) at 2 days, 2 weeks, 1 or 2 months post transplantation. Animal groups ranged from five to eight animals per experimental group as indicated below in order to enable powering of statistical analyses, given the variability of *in vivo* experiments. Histopathological analysis was performed in a blinded manner.

### Histopathology

Animals were anesthetized with a lethal dose of pentobarbital and brains were perfused via the ascending aorta with ice-cold PBS followed by cold 4% paraformaldehyde. Tissues were deep frozen in dry ice, serial 10 μM coronal sections were prepared and immunofluorescent stainings were performed as previously described (Einstein et al., 2003). The following antibodies were used: rabbit anti-glial fibrillary acidic protein (1:200, GFAP, Dako), rat ant-BrdU (1:200, Serotec), rabbit anti-Iba1 (1:250, Wako), rat anti-CD3 (1:200, AbD Serotec) and rat anti-CD200 (1:100, Abcam). Goat anti rat Alexa-fluor555 (1:200), goat anti rabbit Alexa-fluor555 (1:200) and goat anti rabbit Alexa-fluor488 (1:200) were used as secondary antibodies where appropriate. When staining sections containing GFP+ cell grafts, Alexa-fluor555 conjugated secondary antibodies were used. For *in vitro* staining, floating spheres or single cell astrocytes were adhered to 35-mm tissue culture dishes coated with 10 μg/ml poly-D-lysine and 5 μg/ml fibronectin (Sigma). *In vitro* staining was performed as previously described (Einstein et al., 2006a).

### Determination of Graft Survival and Cell Quantification

Every third section was obtained from Bregma +1.0 to −1.0 for striatal grafts, and Bregma −1.0 to −3.0 for hippocampal grafts. Microscopic images (Olympus BX63) were acquired under fixed fluorescence exposure from every 6th section, spanning the entire graft volume. Graft area was measured in each section as number of green (for GFP) or red (for BrdU) pixels above threshold, using ImagePro Plus computerized software. Threshold cutoff was determined as five points above background intensity, based on the software scale ranging 0–225 fluorescence intensity. The graft volume was extrapolated between each two measured sections, and summed up to represent the entire graft volume. We calculated graft survival at 1 or 2 months as a fraction of 2-day old grafts.

Immunofluorescent staining for CD3 and Iba-1 was performed in every 6th section, in adjacent sections to those measuring graft areas. Cell counting was performed manually in a blinded manner adhering to stereological principles. Specifically, only cell bodies that were entirely within or crossing the inclusion (top and right) lines of the frame were counted. For GFP+ grafts that were heavily infiltrated with immune cells, we counted manually in each computer image frame the number of CD3+ or Iba1+ cells per high power (X40) field, residing within the graft area. Mean cell number per field was calculated for each mouse and presented as experimental group average ±SE. For BrdU-labeled grafts (that evoked only minor immune response), the total number of CD3+ cells was counted manually inside the graft area per section. Mean cell number per sectional graft area was calculated for each mouse and presented as experimental group average ±SE.

### Statistical Analysis

The pathological quantifications in transplantation experiments were calculated using Student’s independent (unpaired) samples one tailed *t*-test, according to our null hypothesis of a unidirectional predicted difference between experimental groups. The values are provided as mean ± SE.

## Results

### Allogeneic NPC Graft Rejection Is Dependent on Brain Environment

To examine the role of brain environment in response to allogeneic grafts, we transplanted NPCs. This cellular platform was chosen in view of their neural origin (delivered to their source tissue), their superior ability to survive the transplantation procedure, their therapeutic immune-modulatory properties and their being lead candidates for clinical use.

**Figure 1:**
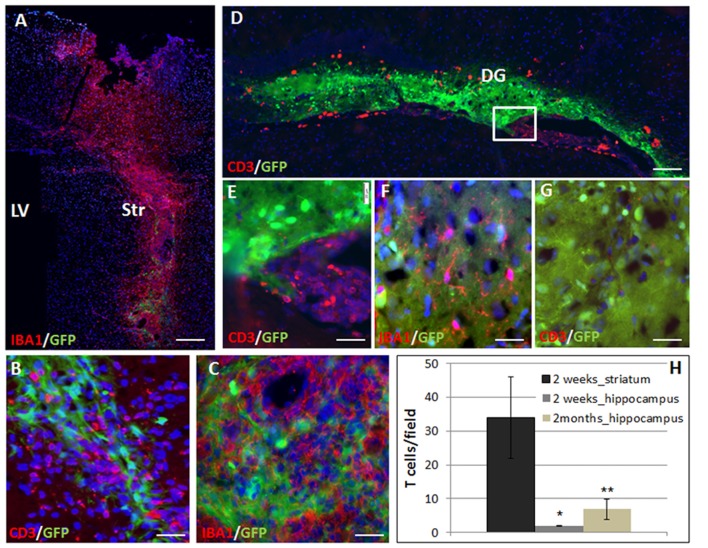
The rejection process toward allogeneic neural precursor cell (NPC) graft is brain region-dependent. Allogeneic green fluorescent protein (GFP)+ NPCs were transplanted to the striatum (*n* = 5) and the hippocampus (*n* = 5) of naïve mice. Two weeks after the transplantation to the striatum **(A)** there was a massive reaction of both CD3+ T cells **(B,H)** and Iba1+ microglia/macrophages **(C)** infiltrating the graft. In contrast, in the hippocampus, ventricular T cell infiltrates were evident (**D**, enlargement **E**) surrounded the hippocampal graft. While microglia/macrophages infiltrated the graft **(F)**, T-cells were essentially restricted and there was only limited T cell infiltration **(G,H)**. Thus the rejection process is region dependent. Str, striatum; LV, lateral ventricle; DG, dentate gyrus. Scale Bar: **(A)** 0.5 mm, **(B,C)** 50 μm, **(D)** 200 μm, **(E–G)** 50 μm. *,**p < 0.05.

Transgenic GFP+ on C57BL6 background NPC spheres were stereotaxically injected into the striatum or hippocampus of BalB/C mice. We studied the rejection process and inflammatory response in the different brain regions by measuring graft size at 2 days and 2 months after transplantation. The immune response was evaluated at 2 weeks and 2 months after transplantation. Given the potentially significant cell death during the transplantation procedure, we chose the 2-days post-transplantation time point as baseline to evaluate graft rejection and survival. Graft survival was therefore calculated as the fraction of the 2 days point at the respective site. Striatal grafts were heavily infiltrated with T cells and macrophages/microglia as early as 2 weeks post-transplantation (Figure 1A: panoramic view, Figures 1B,C: high power field). In contrast, in the hippocampus, there were significant T cell clusters in the ventricle, in proximity to the graft, but only sparse T cell infiltration into the graft at 2 weeks post transplantation (Figure 1D panoramic view, Figures 1E–G—enlargements). The active rejection process in the striatum was evident by finding 34 ± 12 T cells/field, as compared to 1.94 ± 0.17 T cells/field in the hippocampus (Figure 1H).

**Figure 2:**
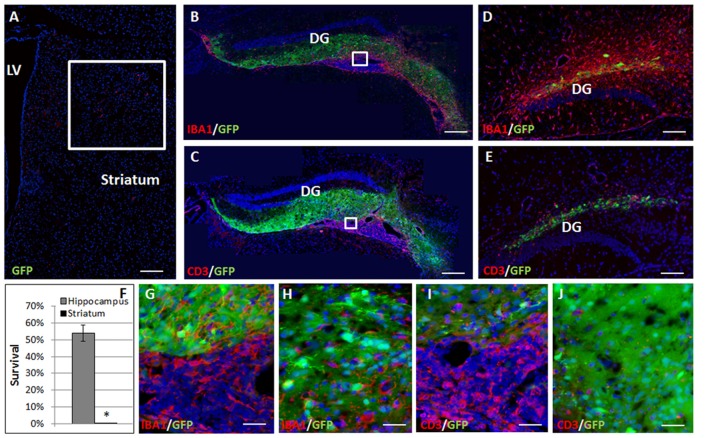
Allogeneic NPC graft rejection is cell and brain region-dependent. Two months after the transplantation there was complete elimination of the graft from the striatum **(A,F)**. In contrast, 2 months after the transplantation 54 ± 4.9% graft survival was detected in the hippocampus **(B,C,F)**. Large ventricular infiltrates of both microglia/macrophages (**B**, enlargement **G**) and T cells (**C**, enlargement **I**) surrounded the hippocampal graft. While microglia/macrophages infiltrated the graft **(H)**, T-cells were essentially restricted and there was only limited T cell infiltration **(J)** minimizing the rejection process. Interestingly, 1 month following astrocyte transplantation to the hippocampus (*n* = 5 mice) massive Iba1+ microglia/macrophages **(D)** and CD3+ T cell **(E)** infiltrations were detected throughout the hippocampus, indicating a strong rejection process. Thus, hippocampal immune privilege is restricted to NPC grafts. Str, striatum; LV, lateral ventricle; DG, dentate gyrus. Scale Bar: **(A–C)** 0.5 mm, **(D,E)** 200 μm, **(G–J)** 50 μm. *p < 0.05.

Complete elimination of the striatal grafts was evident at 2 months post-transplantation (Figure 2A). In contrast, at 2 months after the transplantation 54.41 ± 4.9% graft survival was detected in the hippocampus (Figures 2B,C,F). Large ventricular infiltrates of both microglia/macrophages (Figure 2B, enlargement Figure 2G) and T cells (Figure 2C, enlargement Figure 2I) were observed surrounding the hippocampal grafts. Both low- and high-power microscopy showed that while there was marked macrophages/microglia infiltration into the graft (13.99 ± 2.73 cells/field, Figure 2H), T-cells were essentially restricted outside the graft, and there was limited T cell infiltration (6.87 ± 2.93 cells/field, mostly at the graft edge, Figures 1H, 2J), minimizing the rejection process.

**Figure 3:**
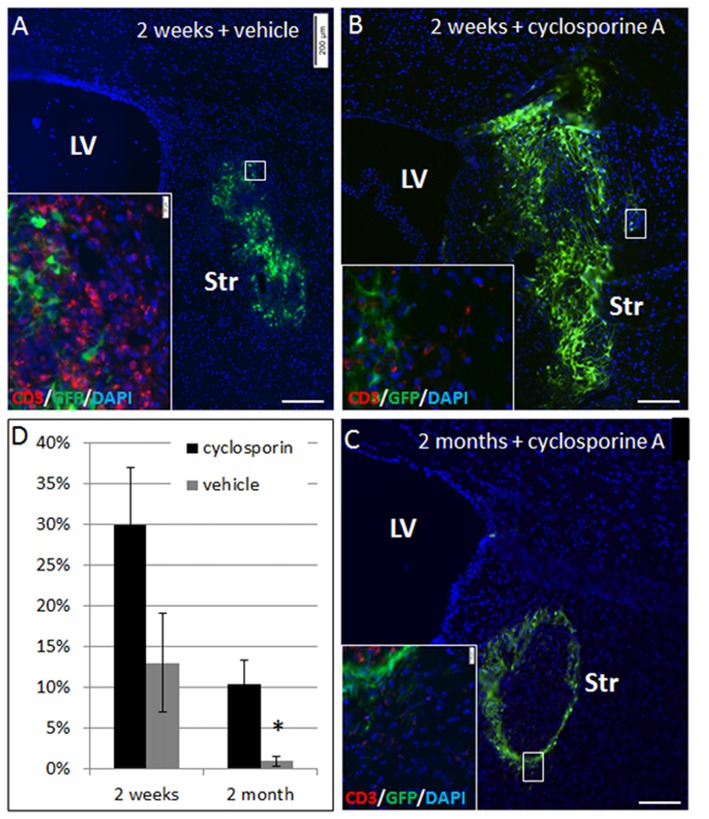
NPC graft elimination is mediated through immune rejection. Allogeneic NPC spheres were transplanted into the striatum, and mice were treated with cyclosporine A to prevent rejection or vehicle. At 2 weeks post-transplantation there was only 13 ± 6% graft survival in vehicle-treated mice (*n* = 5, **A,D**), as compared to 2.5-fold higher graft survival (30 ± 7%) in cyclosporine A –treated mice (*n* = 5, **B,D**). Vehicle treated mice exhibited massive T cell infiltration (**A**, insert), while in cyclosporine A treated mice there was only negligible T cell infiltration (**B**, insert). Two months after the transplantation, cyclosporine treated mice (*n* = 8) showed 10 ± 3% survival **(C)**, with negligible T cell infiltration (**C**, insert), as opposed to <1% graft survival in vehicle treated mice (*n* = 8) **(D)**. Str, striatum; LV, lateral ventricle. Scale Bar: **(A–C)** 200 μm. *p < 0.05.

To ascertain that elimination of NPC grafts from the striatum was due to immune rejection, we compared allogeneic NPC graft survival in Cyclosporine-treated vs. vehicle-treated mice. Striatal grafts in mice that were treated by daily injection with cyclosporine exhibited marked improvement in graft survival at 2 weeks (30 ± 7% vs. 13 ± 6% survival, *p* = 0.08) and at 2 months (10 ± 3% vs. <1%, *p* = 0.02) post-transplantation (Figure 3).

Thus, the immune response is dictated by the CNS environment. Transplanted allogeneic neural precursors are readily infiltrated with T cells and rejected from the striatum, whereas they are partially protected from T cell infiltration and are not rejected from the hippocampus.

### Non-stem Cell Neural Grafts Are Rejected From the Hippocampus

To examine whether the lack of rejection from the hippocampus is dependent on type of transplanted cells, and specifically on their stem cell properties, we examined the fate of transplanted astrocytes. One month after transplantation of GFP+ astrocytes to the hippocampus, there were massive infiltrations of CD3+ T cells (Figure 2D) and Iba1+ microglia/macrophages (Figure 2E) throughout the hippocampus indicating a strong rejection process. Thus, the hippocampal ability to protect the graft from rejection is restricted to NPCs.

### The Role of CD200 Expression on Grafted NPCs in Modulating the Rejection Process in the Striatum

**Figure 4:**
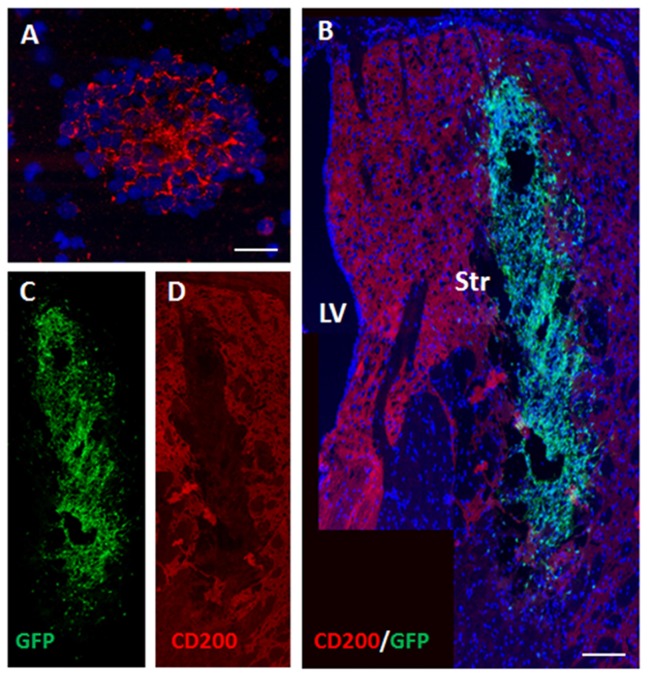
Transplanted NPCs lose the expression of CD200 in the striatum. We examined the expression of CD200 molecule, a “don’t eat me” signal, on NPCs as a possible protector of cell survival. Prior to transplantation, NPC spheres highly expressed CD200 **(A)**. Following transplantation to the striatum (*n* = 5 mice), CD200 expression on grafted cells was reduced (**B**, split color channels in **C,D**). Str, striatum; LV, lateral ventricle. Scale Bar: **(A)** 20 μm, **(B)** 0.5 mm.

CD200 is a cell-surface protein and part of the immune synapse which acts on myeloid- and dendritic CD200-receptors to limit the immune response and protect grafts from rejection. It is also a powerful “don’t eat me” signal to phagocytic cells. We examined the expression of CD200 on NPCs as a possible protector of cell survival. On the day of transplantation NPC spheres highly expressed CD200 (Figure 4A). At 2 days after transplantation to the striatum there was a significant decrease in CD200 expression in grafted NPCs (Figures 4B–D).

**Figure 5:**
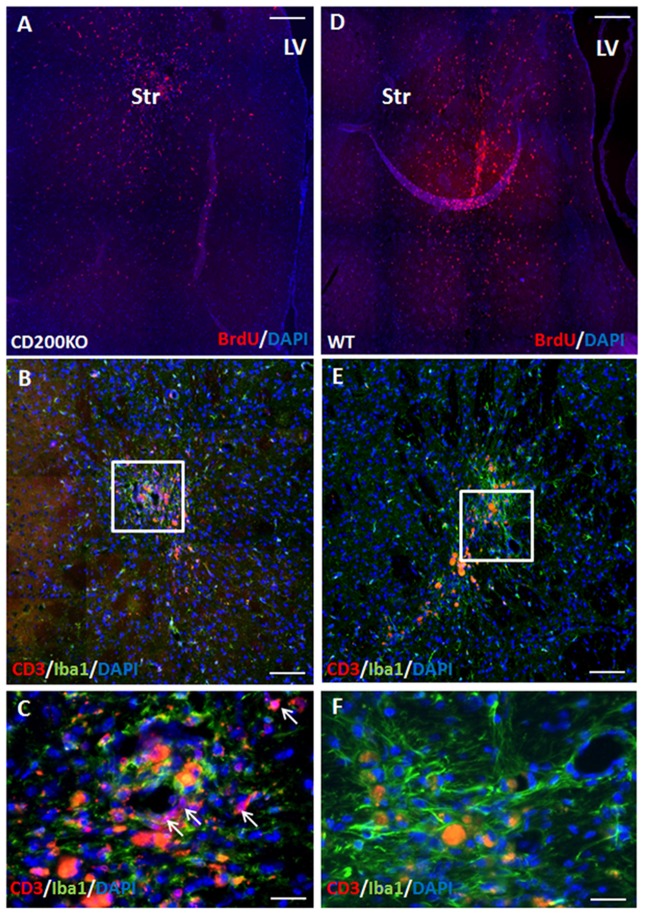
CD200 knock-out NPCs are rejected more rapidly from the striatum. To study whether CD200 expression has a role in protecting striatal grafts from rejection, we transplanted to the striatum BrdU-labeled CD200KO NPCs or wt NPCs (*n* = 7 mice per group). Two months after transplantation, the CD200KO-NPCs **(A)** showed 40% lower survival compared to wt NPCs (**D**, *P* = 0.05, by student *T* test). Examination of the brain immune response revealed strong reaction of microglia/macrophages in both CD200KO and wt transplanted brains (**B,C** enlargement; **E,F** enlargement, respectively). However, T cell infiltration was significantly stronger in striata transplanted with CD200KO NPCs (arrows, **B,C** enlargement). The red auto-fluorescence represents dead grafted cells and was distinguished from CD3 expression in high power field microscopy. Str, striatum; LV, lateral ventricle. Scale Bar: **(A,D)** 0.5 mm, **(B,E)** 100 μm, **(C,F)** 20 μm.

To further study whether CD200 expression has a role in protecting striatal grafts from rejection, we transplanted to the striatum BrdU-labeled NPC spheres grown from CD200 knock-out mice or from wild type mice and compared their survival. As GFP is a highly immunogenic protein and has been associated with aggravated immune response (Fainstein et al., 2013b; Ansari et al., 2016), the use of non-GFP expressing NPCs in this experiment resulted in a slowed rejection process, and enabled the evaluation of the role of CD200 in the rejection process from the striatum. Two months after transplantation, there were 41.2 ± 7.5% less CD200KO-NPCs (*P* = 0.01, Figure 5A) compared to wild-type NPC survival (Figure 5D). Histopathological examination of the immune response towards the striatal grafts revealed a strong reaction of IBA1+ microglia/macrophages in both CD200KO and wild type transplanted brains (Figures 5B,C enlargement; Figures 5E,F enlargement, respectively). However, significantly stronger T-cell infiltration was detected in striata transplanted with CD200KO-NPCs (17.62 ± 7.27/100,000 pixel graft area as compared to wt-NPCs (2.99 ± 0.19/100,000 pixel graft area, *P* = 0.039; Figure 5B, arrows in Figure 5C, enlargement). Thus, lack of CD200 expression facilitates rejection of allogeneic NPCs from the striatum.

### Hippocampal Protection of Allogeneic NPC Grafts Is Not Mediated by CD200 Expression

**Figure 6:**
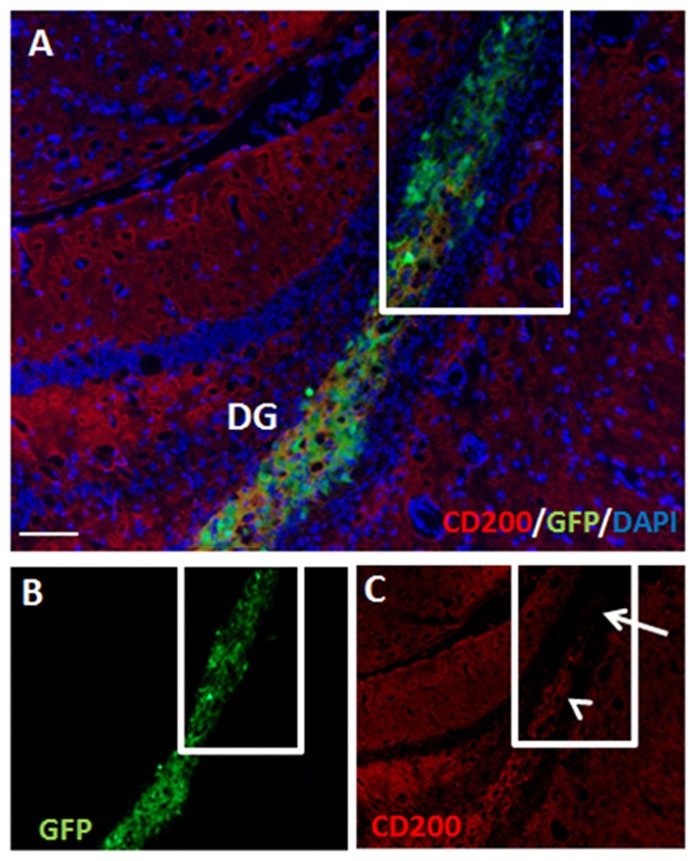
Hippocampal NPC grafts retain partial CD200 expression but lose CD200 expression in extra-hippocampal graft extensions. There was continued partial expression of CD200 by grafted cells at 2 months after transplantation to the hippocampus (**A**, split color channels in **B,C**). Grafted cells which extended out of the hippocampus lost CD200 expression (**C**, arrow). DG, dentate gyrus. Scale Bar: **(A)** 200 μm.

At 2 months post-transplantation, hippocampal NPC grafts retained partial CD200 expression (Figures 6A–C). Furthermore, in parts of the grafts that extended out of the hippocampus, significant loss of CD200 expression was noted (Figure 6C).

**Figure 7:**
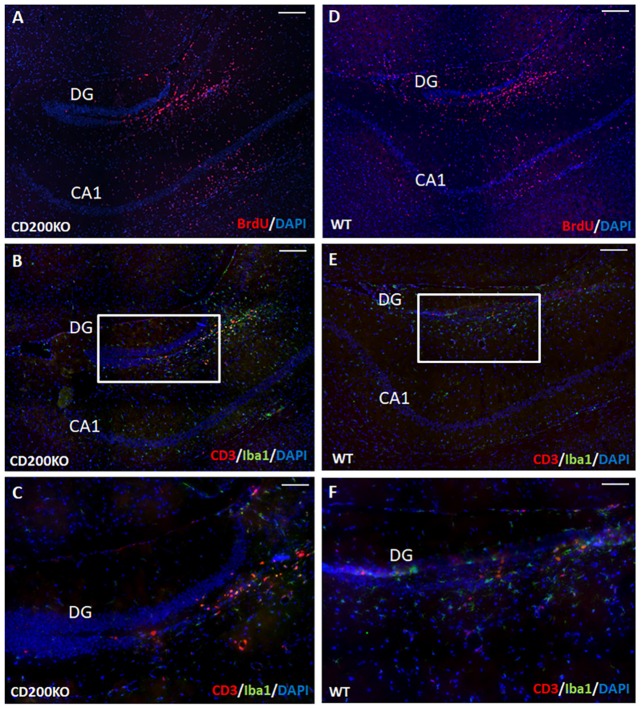
Hippocampal protection of allogeneic NPC grafts is not mediated by CD200 expression. To investigate the hippocampal trait of immune–privilege, we examined the fate of transplanted BrdU-labeled CD200KO NPCs or wt NPCs (*n* = 7 mice per group). No difference was detected in the survival of CD200KO **(A)** vs. wt **(D)** NPCs in the hippocampus (*P* = 0.42 by student *T* test). No significant difference was observed in the minor IBA-1+ and CD3+ immune response toward CD200KO NPC grafts (**B,C** enlargements) and wt NPC grafts (**E,F** enlargement). Thus, hippocampal immune privilege is restricted to NPC grafts, regardless of CD200 expression. The CA1 and dentate gyrus (DG) regions are shown for purpose of orientation. Scale Bar: **(A,B)** and **(D,E)** 300 μm, **(C,F)** 120 μm.

We therefore examined whether the hippocampal protection of transplanted cells from immune rejection is mediated by continued CD200 expression in transplanted NPCs. To that end, we transplanted BrdU-labeled, CD200KO NPCs (Figure 7A) and compared their survival and the immune response they elicited to that of wild type BrdU-labeled NPCs (Figure 7D). No difference was detected in the survival of CD200KO vs. wild-type NPCs in the hippocampus (141,452 ± 22,616 and 135,164 ± 19,609 pixels of BrdU-fluorescence per hippocampus for CD200KO grafts and wild type grafts, respectively, *P* = 0.42). Quantification of T-cell infiltration into the dentate gyrus (DG) at 1 month after transplantation showed no significant difference in the negligible CD3+ T cell response toward the CD200KO as compared to wild type NPC grafts (2.58 ± 0.88/100,000 pixel graft area vs. 3.52 ± 2.12/100,000 pixel graft area respectively, *P* = 0.35, Figures 7B–F). Similarly, there was no difference in IBA-1 staining (data not shown, representative images in Figures 7B,C enlargement; Figures 7E,F enlargement, respectively).

Thus, while the hippocampal environment induces continued expression of CD200 in transplanted NPCs, its expression is dispensable for hippocampal protection of NPC grafts from rejection. Overall, our findings suggest that the hippocampus is a unique brain environment, supporting selective host—NPC graft interactions, to prevent allogeneic NPC graft rejection, regardless of CD200 expression.

## Discussion

We show here that the hippocampus is a partially immune privileged site which protects NPC grafts, as compared to the complete immune-rejection in the Striatum. Hippocampal immune privilege was restricted to transplantation of stem cells.

NPC transplantation has been implied for therapeutic use in various brain disorders, representing both acute and active disease states as well as chronic and quiescent conditions. The fate of grafted cells is clearly dependent on both cell type and host environment. Transplantation into the acutely-injured brain has usually resulted in a favorable graft fate. For example, induction of acute neuro-inflammation by Quinolinic acid improved the survival of allogeneic striatal grafts, as compared to survival in non-lesioned, intact host brain (Duan et al., 1998). Thus, the acutely injured brain environment cannot be used to evaluate and compare the basic immunogenic properties of different host brain regions. In order to evaluate these properties, we performed here allogeneic NPC transplantations to distinct regions of the intact brain.

CNS immune privilege has been traditionally attributed to the BBB. The effective restriction of T cell infiltration from hippocampal grafts in spite of BBB breakage by the surgical procedure highlights the uniqueness of hippocampal environment. These results suggest that additional tissue properties contribute to hippocampal immune-privilege. Our study suggests also that the immune-privileged property of the hippocampus is not universal but may be restricted to transplants with stem cell identity. It is well-established that transplanted NPCs possess powerful therapeutic immune-regulatory properties *in vivo* (Pluchino et al., 2003, 2005; Einstein et al., 2006b, 2007; Aharonowiz et al., 2008). Indeed, xenogeneic NPC grafts were less prone to rejection than primary tissue grafts (Armstrong et al., 2001). However, we have shown that transplanted NPCs lose their immune-modulatory properties with time following introduction into the naïve striatum, enabling, eventually, their rejection (Fainstein et al., 2013b). Although Astrocytes were shown to suppress alloantigen elicited immune response *in vitro* (Akesson et al., 2009), they do not possess such functional properties *in vivo* (Einstein et al., 2003). Moreover, astrocytes can promote immune responses following CNS injury *in vivo*, acting like macrophages, capable of phagocytosis and recruitment of immune cells (Liddelow and Hoyer, 2016). Indeed, our findings show that Astrocyte grafts were readily infiltrated with T cells and rejected from the hippocampus, whereas NPC grafts precluded T-cell infiltration and survived. The marked difference between the strong rejection of astrocytes vs. the survival of NPCs might point towards the existence of graft-host interaction that dictates the functional state of transplanted cells. Notably, the DG of the hippocampus is a site of continued neurogenesis and a physiological niche for NPCs (Gonçalves et al., 2016), which may therefore maintain their stem cell properties. Our grafts were placed mainly in the DG, and it is not clear to what extent the protective effect is applied to the entire hippocampus. The present study suggests that the hippocampal milieu might enable the continued expression of functional immune-regulatory properties by transplanted NPCs that protect them from rejection. In agreement, we have previously shown marked regional differences in host tissue support of syngeneic NPC graft survival. In that study transplanted syngeneic NPCs survived extensively in the intact hippocampus but survived significantly less following introduction to the intact striatum (Fainstein et al., 2013a). These further highlight the concept of NPC grafts as a dynamic cell population, the fate of which is not determined solely by their original pre-transplant properties, but also by host CNS environmental influences.

To further point out at the unique properties of the hippocampal milieu, we examined the role of CD200 in determining graft fate. The role of CD200 in protecting transplanted cells is supported by the multiple functions of this protein. Cell-surface expressed and secreted CD200 act as a potent suppressor of brain innate immunity (Hernangomez et al., 2014). Moreover, CD200 plays a central role in preventing T cell influx into the ischemic hind limb in a mouse model of ischemic injury (van den Borne et al., 2014). CD200 expression is important in limiting immune rejection processes. For example, over-expression of CD200 improved skin allograft survival (Wong et al., 2012; Yu et al., 2013; Gorczynski et al., 2014), and was shown to mediate the ability of both myeloid (Conticello et al., 2013) and CNS-derived tumors (Moertel et al., 2014) to evade immune surveillance. Furthermore, CD200 expression may provide cells with immune-suppressive properties, as CD200 level of expression on fetal- vs. maternal-derived placental mesenchymal stem cells correlated with their immunosuppressive properties (Zhu et al., 2014). As expected, our findings suggest an important role for CD200 expression by transplanted NPCs in restricting T cell infiltration into the graft and limiting graft rejection from the striatum. The loss of CD200 expression on transplanted NPCs was associated with enhanced T cell infiltration into the graft and expedited immune rejection from the striatum. In contrast, CD200 expression was dispensable for protecting hippocampal NPC grafts from rejection, again exemplifying the uniqueness of the hippocampus.

In sum, we observed marked regional differences in the immune response in the brain, wherein the hippocampus acts as an immune sanctuary for allogeneic NPC grafts. In view of the selectivity in hippocampal immune privilege, occurring only with NPC grafts, we hypothesize that the hippocampus potentiates the continued expression of NPC native immune-modulatory function. Therefore the hippocampus enables transplanted neural precursor/stem cells to protect themselves from rejection.

## Author Contributions

NF: collection and/or assembly of data, provision of study material or patients, data analysis and interpretation, manuscript preparation. TB-H: conception and design, manuscript preparation, final approval of the manuscript.

## Conflict of Interest Statement

The authors declare that the research was conducted in the absence of any commercial or financial relationships that could be construed as a potential conflict of interest.
